# Serum ferritin in Yemeni patients with sickle cell anemia: Association with sociodemographic factors and hematological profiles

**DOI:** 10.1371/journal.pone.0350016

**Published:** 2026-05-27

**Authors:** Abdulsalam Al-Shami, Lutfi A. S. Al-Maktari, Lina Qasem, Khalid Aldhorae, Shimaa AL-Khaship

**Affiliations:** 1 Department of Clinical Biochemistry, Faculty of Medicine and Health Sciences, Taiz University, Taiz, Yemen; 2 Department of Hematology, Faculty of Medicine and Health Sciences, Sana’a University, Sana’a, Yemen; 3 Department of Medical Biochemistry, Faculty of Medicine and Health Sciences, Amran University, Amran, Yemen; 4 Faculty of Dentistry, University of Ibn al-Nafis for Medical Sciences, Sana’a, Yemen; The Ohio State University, UNITED STATES OF AMERICA

## Abstract

**Background:**

Iron overload remains a critical complication secondary to transfusional therapy and altered iron metabolism in patients with sickle cell anemia (SCA). Hyperferritinemia often serves as a surrogate marker for iron overload; however, as an acute-phase reactant, it may be elevated due to chronic inflammation, infection, or liver disease, potentially overestimating true iron stores. In Yemen, the burden of SCA is high, yet local data on the frequency of hyperferritinemia are scarce. This study aimed to describe the distribution of serum ferritin (SF) levels and the proportion of SCA patients with hyperferritinemia among adolescents and young adults attending the YSTH clinic in Sana’a, and to explore associations with hematological, and sociodemographic parameters.

**Methods:**

A prospective cross-sectional study was conducted between June and December 2024, involving 80 participants (aged ≥ 15 years). The data were analyzed using SPSS version 26. Due to the non-normal distribution and high dispersion of SF, the Median (IQR) and Spearman correlation were utilized. Bivariate analysis calculated crude odds ratio at (95% CI) to identify predictor of elevated SF. Statistical power was limited by the sample size (*N* = 80) to detect weak associations.

**Results:**

The study enrolled 80 SCA patients with a mean (±SD) age of 18.5 (±3.69) years, of whom 65% were males and 35% were females. The median (IQR) SF level was 1645 ng/mL (414–3525), with values ranging from 77.1 to 4500.0 ng/mL. Approximately 85% of patients exhibited elevated SF level (≥300 ng/mL). While red cell indices did not significantly predict the risk of hyperferritinemia, a significant positive correlation was observed between SF levels and white blood cell count (*p* = 0.03), suggesting a possible link with the inflammatory state. No significant associations were found between sociodemographic factors and high SF levels.

**Conclusion:**

A high prevalence (85%) of hyperferritinemia *(*≥ *300 ng/mL)* was observed among SCA patients attending the YSTH in Sana’a, Yemen, suggesting probable iron overload. While findings support routine SF screening but highlight the need for future studies incorporating inflammatory markers (e.g., CRP) and systematic transfusion histories to definitively quantify the iron overload burden in this population.

## Introduction

Sickle-cell anemia (SCA) refers to a group of genetic disorders characterized by the inheritance of two abnormal copies of hemoglobin genes, known as homozygous hemoglobin S (HbSS). This leads to the formation of rigid, sickle-shaped red blood cells that obstruct blood flow. SCA is caused by a point mutation at the sixth position of β**-**globin gene on chromosome 11, where glutamic acid is replaced by valine (GAG → GTG) [1,2]. According to estimates from the World Health Organization (WHO), 7% of the global population carries an abnormal Hb gene, with 300,000–500,000 infants born annually with hemoglobinopathies. Of these, sickle cell syndromes are the most frequent, accounting for 70% of cases worldwide [3]. In Yemen, approximately 4% of the population carries an abnormal HbS gene, with a predicted birth incidence of 20 per 10,000 each year [4].

In addition to chronic hemolytic anemia, SCA causes severe vaso-occlusive crises (VOC), the most prevalent characteristic of SCA. These occur when sickled red blood cells block microvasculature, triggering inflammation and tissue ischemia. VOCs are responsible for a range of SCA complications, including stroke, acute chest syndrome, leg ulcers, nephropathy, and retinopathy. Chronic complications, such as progressive organ damage, often manifest years after disease onset [5,6].

It is generally believed that patients with SCA may accumulate excess body iron predominantly due to repeated blood transfusions, while chronic hemolysis and increased gastrointestinal iron absorption modulate iron distribution and regulation rather than directly increasing total iron load in non-transfused individuals [7]. However, some studies suggest that iron deficiency may occur in SCA, particularly in patients who have not received transfusions [8,9]. Since both iron deficiency and iron overload significantly worsen clinical outcomes, early identification and monitoring of iron status are critical [7,10]. Hence, Effective screening enhances immediately medical interventions, reducing severe complications and mortality [2,11,12].

In patients with SCA, the commonly used laboratory tests for iron status assessment are serum ferritin (SF), total iron binding capacity (TIBC), and transferrin saturation. Of these parameters, serum ferritin is the most principal test used not only for assessing iron deficiency but is also considered as a good marker for iron overload [13]. Nonetheless, because ferritin is an acute-phase reactant, its elevation may reflect underlying inflammation, infection, or hepatic dysfunction—all common in SCA—and should ideally be interpreted in conjunction with inflammatory markers and organ-specific imaging (e.g., magnetic resonance imaging [MRI]).

Little is known of the frequency and risk factors associated with hyperferritinemia in the Middle East among patients with SCA; in Yemen, to our understanding, remain limited. Therefore, this study aimed to describe the distribution of serum ferritin levels and the proportion of SCA patients with elevated ferritin among adolescents and young adults at the Yemen Society for Thalassemia and Genetic Blood Disorders (YSTH), and to explore associations with hematological, and sociodemographic parameters.

### Methods study design and setting

A prospective cross-sectional study was conducted among patients with SCA attending the sickle cell clinic of the Yemeni Society for Thalassemia and Genetic Blood Disorders (YSTH), Sana’a, Yemen, between June 2, 2024, and December 31, 2024. YSTH is a non-profit humanitarian association established in 2000 to provide lifelong care for patients with inherited hemoglobinopathies across Yemen.

#### Study population.

The study included 80 patients (aged ≥15 years) diagnosed with SCA and receiving regular care at YSTH. Written informed consent was obtained from all participants or their legal guardians prior to recruitment. Exclusion criteria included: age < 15 years, sickle cell trait (HbAS), hemoglobin phenotype SC, current iron chelation therapy, history of chronic liver or renal diseases, blood transfusion within the past three months, or refusal to provide informed consent.

#### Sample size.

The sample size was calculated using the following formula: n = Z^2^ P q/d^2^. Where: n = minimum sample size, Z = 1.96 (95% confidence interval), P = expected frequency of hyperferritinemia (28.5% based on a previous study [14]), q = 1-P (0.715), and d = degree of precision (0.10). The minimum estimated sample size was 78; thus, our cohort of 80 patients met the requirement for descriptive analysis.

#### Data collection.

Sociodemographic data (age, sex, marital status, and family history) were collected via a structured questionnaire. Clinical data in the last 12 months, including joint pain, limb swelling, splenomegaly, and fever were recorded. Venous blood (4 ml) was collected by trained phlebotomists; 2 ml was placed in an ethylenediaminetetraacetic acid (EDTA) tube for hematological analysis, and 2 ml was placed in a serum separator (SS) tube for ferritin testing.

#### Laboratory investigations.

Complete blood count (CBC), including hemoglobin (Hb), white blood cell count (WBC ct) and differential count, platelet count (PLT ct), and red cell indices [mean corpuscular volume (MCV), mean corpuscular hemoglobin (MCH), mean corpuscular hemoglobin concentration (MCHC)], Red Cell Distribution Width (RDW), were measured using an automated hematology analyzer (Sysmex KX-21N, Sysmex, Kobe, Japan). Serum ferritin level was determined using the chemiluminescence immunoassay (CLIA) method on a Cobas e 411(Roche Diagnostics GmbH, Penzberg, Germany). SF was the primary marker utilized for iron status; no imaging (e.g., MRI or FerriScan) or additional iron markers (e.g., TIBC, transferrin saturation) were available for this cohort. Results were interpreted using the manufacturer’s reference range (20–300 ng/mL); however, no systematic correction for inflammatory markers(e.g., C-reactive protein [CRP]) was performed, as these were not available. Samples result exceeding 3000 ng/mL were diluted to ensure accuracy.

### Ethical statement

The study was conducted in accordance with the Declaration of Helsinki. Ethical approval was obtained from the Research Ethics Committee of the Faculty of Medicine and Health Sciences, Taiz University, Yemen (Ref: ECTU2024/12–1). Written informed consent was obtained from all participants or their legal guardians after a full explanation of the purpose of the study. Data confidentiality and participant privacy were strictly maintained.

### Data analysis

Data were analyzed using the Statistical Package for Social Sciences Software (SPSS) version 26 (SPSS Inc., Chicago, IL, USA). Descriptive statistics included were frequency and percentage for categorical variables, while continuous variables were expressed as mean ± standard deviation (SD) or median and interquartile range (IQR) given the non-normal distribution of ferritin. Bivariate analysis was performed to calculate the crude odds ratio at 95% CI between the dependent variable and categorical predictors. Given the sample size (*N* = 80), the study has limited power to detect weak or moderate associations. Ferritin was analyzed both as a continuous variable and categorically (< 300, 300–1000, and > 1000 ng/mL) to assess clinical relevance. Correlations between serum ferritin and hematological parameters were performed using the Spearman correlation test. Statistical significance was set at *p* < 0.05.

## Results

### Demographic characteristics of enrolled participants

A total of 80 patients with SCA were enrolled. As shown in Table 1, 52 (65%) participants were males and 28 (35%) were females (ratio 1.9:1). The mean (±SD) age was 18.5(±3.69) years. The majority of patients (n = 56,70%) were aged 15–19 years (< 20 years), while 24 (30%) were aged 20 years or older. Most of participants were single (74; 92.5%), and 68 (85%) reported a positive family history of SCA.

**Table 1:** Demographic and clinical characteristics of study participants (*N* = 80).

Variable	Frequency (n)	Percentage (%)
Age (years)	Mean ± SD (Min–Max)	18.5 ± 3.69 (15–25)
< 20 (15–19)	56	70.0
≥ 20 (20–25)	24	30.0
Gender		
Male	52	65.0
Female	28	35.0
Marital status		
Single	74	92.5
Married	6	7.5
Family History		
Yes	68	85.0
No	12	15.0
Clinical characteristics in the last 12 months		
Severe joint pain	80	100.0
Limb swelling	44	55.0
Fever	18	23.0
Splenomegaly	12	15.0

Note: Results are expressed as frequency (*n*) and percentage (%). SD: standard deviation. Min: minimum; Max: maximum.

Clinical evaluation revealed that all participants (100%, n=80/80) experienced joint pain, with other findings including limb swelling (55%, n=44/80), fever (23%, n=18/80), and splenomegaly (15%, n=12/80) within the last 12 months.‌‌

### Serum ferritin levels and hematological indices

Due to the non-normal distribution of serum ferritin (SF), levels are reported as median and interquartile range (IQR). The median SF concentration was 1645 ng/mL (IQR: 414–3525), with a total range of 77.1 to 4500 ng/mL) (Table 2). Based on SF thresholds, 12 (15%, *n* = 12/80) had normal levels < 300 ng/mL, 16 (20%, *n* = 16/80) had levels between 300–1000 ng/mL and 52 (65%, *n* = 52/80) participants had severe elevations exceeding 1000 ng/mL. Collectively, 68 (85%, *n* = 68/80) participants exhibited hyperferritinemia (≥ 300 ng/mL).

**Table 2:** Serum ferritin levels and hematological profile of study participants (*N* = 80).

Variable			
	Frequency (%)	Minimum	Maximum
**Serum Ferritin levels (ng/mL)**			
**Mean ± SD/ Median (IQR)**	1910 ± 1432/1645 (414-3525)	77.1	4500
< 300	12 (15)	—	—
300–1000	16 (20)	—	
>1000	52 (65)	—	—
**Hematological Profile**	Mean ± SD/ Median ((IQR)	Minimum	Maximum
Hb (g/dL)^a^	8.7 ± 1.0	6.5	10.9
RBC **ct** (× 10^6^ / µL)^a^	3.0 ± 0.9	1.9	4.9
WBC **ct** (× 10^3^ / µL)^a^	10.3 ± 3.5	3.0	18.8
HCT %^a^	27.2 ± 3.2	18.8	35.1
MCV (fL)^a^	91.9 ± 12.1	72.2	140.9
MCH (pg)^a^	30.3 ± 6.8	21.5	77.4
MCHC (g/dL)^a^	32.3 ± 1.5	23.5	34.5
RDW (fL)^a^	22.4 ± 3.6	16.3	31.8
PLT **ct** (× 10^3^/ µL)^b^	464 (105–1808)	—	—

Note: Results are expressed as frequency and percentage (*n*,%), ^a^ Mean ±SD, ^b^ Median (IQR). Hb, Hemoglobin; RBC ct, Red Blood Cell Count; HCT, Hematocrit; MCV, Mean Cell Volume; MCH, Mean Cell Hemoglobin; MCHC, Mean Cell Hemoglobin Concentration; RDW, Red Cell Distribution Width; PLT ct, Platelet Count; *N*, Number of participants; g/dL, Gram per deciliter; μL, microliter; fL, Femtoliter; pg, Picogram.

The hematological profile is summarized in Table 2. Mean (±SD) values for Hb, RBC, and HCT were 8.7 (±1.0) g/dL, 3.01(±0.9) ×10^6^ /µL, and 27.15 (±3.2)%, respectively. Regarding red cell indices, the mean, MCV was 91.9 (±12.1) fL and MCH was 30.3 (±6.8) pg. The median platelet was 464 × 10^3^/µl (IQR:105–1808). Comparison of indices between patients with normal SF (<300 ng/mL) and high SF (≥ 300 ng/mL) showed no statistically significant differences in Hb, MCV, or MCH (*p* = 0.72, 0.32, and 0.44, respectively; Table 3).

**Table 3:** Comparison of red blood cell indices of the SCA patients with normal and high serum ferritin levels (*N* = 80).

	Serum Ferritin levels (ng/mL)		
Red blood cells indices	< 300(n = 12)	≥ 300 (n = 68)	*p*-value
Hb (g/dL)	8.8 ± 1.18	8.7 ± 0.95	0.72
MCV (fL)	88.7 ± 6.75	92.5 ± 12.71	0.32
MCH (pg)	28.9 ± 2.63	30.5 ± 7.28	0.44

Note:Results are expressed as mean ±SD. Statistically significant difference *p*-value <0.05

Furthermore, we compared the mean values of red blood cells indices between SCA patients with normal SF (< 300 ng/mL) and high SF (≥ 300 ng/mL). As shown in Table 3, there was no statistically significant differences between two groups regarding mean Hb, MCV, or MCH (*p* = 0.72, 0.32, and 0.44, respectively).

### Factors associated with hyperferritinemia

As shown in Table 4 and 5, neither age nor sex were significantly associated with SF levels. Although mean SF value was higher in the older age group (≥ 20 years: 2010 ± 1519) compared to the younger group (< 20 years: 1868 ± 1406), this difference was not statistically significant (*p* = 0.68). Similarly, while males exhibited a higher mean SF (2111 ± 1409 ng/mL) (1539 ± 1426 ng/mL), this difference did not reach statistical significance (*p* = 0.08, Table 4).

**Table 4:** Correlation between serum ferritin levels, age and sex in study participants with SCA (N = 80).

Variable	Frequency(%)	Serum ferritin levels (ng/mL)	*p*-value
Age			
< 20	56 (70)	1868 ± 1406	0.68
≥ 20	24 (30)	2010 ± 1519	
Sex			
Male	52 (65)	2111 ± 1409	0.08
Female	28 (35)	1539 ± 1426	

Note: Results are expressed as frequency, percentage (*n*,%), and mean ±SD. Independent t test*, p* < 0.05 was considered to be as statistically significant.

**Table 5:** Bivariate analysis of factors associated with high ferritin levels among patients with SCA (*N* = 80).

Variable	Serum Ferritin levels (ng/mL)			
	< 300(n = 12)	≥ 300 (n = 68)	OR (95% CI)	*p*-value
Age (years)				
< 20	7(15)	40 (85)	0.98 (0.28-3.40)	0.97
≥ 20	5(15)	28 (85)		
Sex				
Male	5(10)	47(90)	0.31(0.09-1.12)	0.06
Female	7(25)	21(75)		

* Results are expressed as frequency and percentage,*n*(%).Statistically significant difference *p*-value <0.05. SCA, sickle cell anemia; OR, odds ratio; CI, Confidence Interval.

Bivariate analysis confirmed that age (*OR*: 0.98; *p* = 0.97) and sex (*OR*: 0.31; *p* = 0.06) were not significant predictors of hyperferritinemia in this cohort (Table 5).

### Correlations with hematological parameters

Using the non-parametric Spearman correlation test, a statistically significant positive correlation was observed between SF levels and the WBC count (ρ = 0.24, *p* = 0.03). No other significant correlations were found between SF and hematological parameters, including Hb, MCV, or RDW (Table 6).

**Table 6:** Correlation of red blood cell indices, white blood cells and hemoglobin with serum ferritin Levels among SCA patients (*N* = 80).

Variable	Spearman (ρ)	p-value
Hb	−0.06	0.58
MCV	−0.09	0.41
MCH	−0.11	0.31
MCHC	−0.11	0.29
RDW	0.01	0.89
Hct	0.01	0.87
WBC	0.24	0.03*

Note: *p* < 0.05 considered as significant value. ρ: Spearman’s correlation coefficient.

## Discussion

This study provides the first data on serum ferritin (SF) levels among SCA patients in YSTH, Sana’a, Yemen, where 85% of the cohort exhibited hyperferritinemia (SF ≥ 300 ng/mL). Notably, 65% of patients had levels exceeding 1000 ng/mL (Table 2). While these proportions align with findings from Kenya (70.5%) [15], they are significantly higher than those reported in Congo (27%) [16] and Nigeria (33%) [17]. However, comparison of hyperferritinemia prevalence across studies is hampered by the lack of systematic transfusion history in our cohort, including the cumulative number of units received and transfusion regimens, which strongly influence iron burden.

In light of these observations, the elevated SF levels in our patients may be partly explained by chronic hemolysis from recurrent infection, inflammation, hepatocellular disease, increased gastrointestinal absorption of iron and hemochromatosis [13,15,18,19], which were not examined in our study. Given that ferritin functions both as an iron storage marker and an acute-phase reactant, its elevation in patients with chronic diseases may reflect underlying inflammatory processes rather than true iron overload [15,20]. So, although, high serum ferritin levels in SCA patients may be primarily an index of their iron stores accumulation, it may be secondary to chronic inflammation [17,20]. The significant positive correlation observed in our study between SF levels and white blood cell (WBC) count (***ρ*** = 0.24, *p* = 0.03) further supports this possibility, as elevated WBC counts often signal an inflammatory state. While high ferritin levels were observed, this study documents hyperferritinemia and can only infer possible iron overload, which may be confounded by chronic inflammation, infection, or liver disease—all frequent in SCA. A key methodological limitation is the lack of concomitant measurement of CRP, interleukin-6 (IL-6), and liver function tests. Strengthening future research by including these markers, alongside additional iron markers (e.g., TIBC, transferrin saturation) or organ-iron assessment (MRI), is a priority to distinguish between inflammatory responses and true iron accumulation.

Furthermore, we observed a male predominance (65.0%), consistent with studies by Patel et al. [21], Rao et al. [22] and Alsuliman et al. [23]. However, the higher mean SF in males (2111 ng/mL) compared to females (1539 ng/mL) was not statistically significant (*p* = 0.08). Similarly, SF levels appeared higher in patients ≥ 20 years, yet this also lacked significance (*p* = 0.68), corroborating evidence that SF may not be a strictly age-dependent function in SCA populations [15–17]. The very wide dispersion of ferritin values (mean 1910 ± 1432 ng/mL with a median (IQR) of 1645 (414–3525); range (77.1–4500) probably reflects heterogeneous transfusion exposure and inflammatory burden across patients—rather than biological sex or chronological age. Without reliable transfusion data, it is difficult to disentangle these contributions. This high variability further limits our ability to identify robust determinants of high ferritin in this specific population.

Although individual transfusion histories could not be reliably reconstructed due to poor documentation and patient recall, future studies should explore institutional transfusion records (e.g., blood bank logs) to estimate average annual transfusion exposure per SCA patient, which would substantially improve the interpretation of ferritin levels. Nevertheless, some studies showed that a past history and number of hemotransfusion units were not significantly associated with serum ferritin level [13,17]. On the other hand, previous studies have demonstrated that, irrespective of number of blood units transfused in last years, SCA patients who had been hemotransfused 1–3 units or more during the year, experienced elevated SF level [1,16,17].

The mean Hb level in our cohort (8.7 g/dL) is comparable to findings in India and Ghana (8.09–8.47 g/dL) [24–26], yet slightly higher than the 7.73 g/dL reported by Rao et al. [22]. These levels are notably lower than those in high-resource settings like the United States (9.1 g/dL), where patients typically have better access to hydroxyurea therapy, comprehensive care, and nutritional support [27]. The persistently low Hb in our population likely reflects the synergistic impact of chronic hemolysis, frequent infections, and systemic nutritional deficiencies [7,8,20]. These clinical challenges are further exacerbated by the ongoing conflict and economic collapse in Yemen since 2015, which has severely limited the availability of routine screening and specialized SCA management, complicating the iron-status profile.

Regarding red blood cell indices, our findings of no significant differences in Hb, RBC, MCH, or MCHC among SCA patients are consistent with data from Al-Khalidi and Ghazzay [28]. While the mean cell volume (MCV) in our cohort aligns with several regional reports [24,25], it remains lower than values observed by Jadhav et al. [29]. Elevated MCV in this population is traditionally attributed to accelerated erythropoiesis secondary to chronic hemolysis and folate deficiency [22], both of which are prevalent in the Yemeni clinical context. Notably, we found no significant correlation between SF levels and RBC indices (Hb, MCV, MCHC). This lack of association further suggests that in Yemeni SCA patients, SF levels are driven more by transfusion events and inflammatory status than by the immediate hematological profile or red cell morphology.

This study has several limitations and strengths that should be considered when interpreting its findings. First, the study was conducted at a single health facility, which may limit the generalizability of our findings to the broader population of SCA patients in Yemen. Second, because serum ferritin is an acute-phase reactant, levels can be influenced by factors other than iron overload, such as infection, chronic inflammation, or hepatic dysfunction. As our study did not include inflammatory markers (e.g., CRP), we cannot definitively exclude these as contributing factors to the observed hyperferritinemia. Finally, due to a poor medical recording in the region, systematic data on lifetime transfusion history and frequency were unavailable. Consequently, we were unable to statistically correlate serum ferritin levels with the total number of hemotransfusions, which is a primary driver of secondary iron overload.

## Conclusion

Our study demonstrates a high burden of hyperferritinemia among SCA patients at this centre, even in a setting where chronic hemotransfusion programs are not standard and transfusions are provided only when clinically indicated. These findings suggest that iron accumulation or chronic inflammatory states are highly prevalent in this population.

Collectively, our results highlight the clinical utility of serum ferritin as non-invasive and cost-effective first-line screening tool Identifying elevated ferritin levels early is essential before initiating either iron chelation or iron supplementation, as it helps mitigate the risk of iron-mediated organ damage. Furthermore, the lack of significant correlation between red cell indices and ferritin levels confirms that traditional hematological markers are insufficient for assessing iron status in SCA. While serum ferritin is a valuable screening marker, its role as an acute-phase reactant remains a key limitation, as evidenced by its significant correlation with WBC count in our cohort. Therefore, we reinforce the need for incorporating inflammatory markers (e.g., CRP) into routine practice and advocate for the future availability of advanced diagnostic tools, such as Magnetic resonance imaging (MRI) or FerriScan® (R2-MRI), as the gold standard for accurately quantifying iron burden in the liver and heart within the Yemeni healthcare system.

## Supporting information

S1 Dataset Minimal underlying dataset. This Excel file contains the anonymized hematological and ferritin data for the study population.
(XLSX)

## Abbreviations

CBC: complete blood count
CI: confidence interval
CLIA: chemiluminescence immunoassay
EDTA: ethylenediaminetetraacetic acid
HbS: hemoglobin S
IL-6: interleukin-6
IQR: interquartile range
MCH: mean corpuscular hemoglobin
MCHC: mean corpuscular hemoglobin concentration
MCV: mean corpuscular volume
MRI: magnetic resonance imaging
OR: odds ratio
PLT ct: platelet count
RBC ct: red blood cell count
RDW: red cell distribution width
SCA: sickle-cell anemia
SD: standard deviation
SF: serum ferritin
SPSS: statistical package for the social sciences
TIBC: total iron binding capacity
VTE: vaso-occlusive crises and venous thromboembolism
WBC ct: white blood cell count
YSTH: Yemeni society for Thalassemia and genetic blood disorders
